# Supplementary material for: Salivary levels of Streptococcus mutans and Lactobacilli and other salivary indices in patients wearing clear aligners versus fixed orthodontic appliances: An observational study
Source: PLoS One. 2020 Apr 24;15(4):e0228798. doi: 10.1371/journal.pone.0228798 (PMC7182227; doi:10.1371/journal.pone.0228798)
Supplement: Attachment — Submitted filename: Peer review PLOS ONE 19-21131.docx [file pone.0228798.s002.docx]

This manuscript described to investigate salivary levels of Streptococcus mutans (S.mutans) and Lactobacilli, and other salivary indices in patients wearing clear aligners (CA) versus multibrackets orthodontic appliances (MB).The authors determined Plaque index (PI), salivary flow, buffering power of saliva, and

salivary levels of S.Mutans and Lactobacilli were evaluated before the beginning of the orthodontic treatment (t0), and after 3 months (t1) and 6 months (t2) from the beginning. The authors recruited 80 subjects with equal numbers of Mb and CA treatments.

Overall this study is an interesting area of investigation; however, there were some limitations and questions that the author should clarify for the comprehensive understanding of the manuscript.

1. Please provide the time of saliva collection since it affects the composition and flow of saliva.

2. Please discuss the association between number of bacteria and incidence of decalcification in the discussion.

3. Normally the orthodontic treatment will take about 12 months, would the author follow up after 6 months. If so, would the author observe any difference or trend of bacteria level?

4. Would the author explain the randomization process of subjects?

Thank you for the opportunity to review this manuscript.
