# Supplementary material for: Salivary levels of Streptococcus mutans and Lactobacilli and other salivary indices in patients wearing clear aligners versus fixed orthodontic appliances: An observational study
Source: PLoS One. 2020 Apr 24;15(4):e0228798. doi: 10.1371/journal.pone.0228798 (PMC7182227; doi:10.1371/journal.pone.0228798)
Supplement: Attachment — Submitted filename: R1.docx [file pone.0228798.s003.docx]

Reviewer #1: Thank you for your submission

**A few issues - firstly, the English is not easy to read, so i suggest you recruit a native English speaker knowledgeable in cariology or microbiology**

**R:** The manuscript was fully proofreaded by a native English speaker dentist

**The term 'patient' should be avoided and replaced with 'participant'**

**R:** thanks for the suggestion. It was changed all over the manuscript

**Streptococcus mutans in italics or S. mutans (space between S. and mutans) should be used - you have a variety of iterations of these**

**R:** thanks for the suggestion. It was changed all over the manuscript

**On page 4, you mention 'caries' incidence increases as well as white spot lesions - white spot lesions are carious lesions - please correct terminology - maybe refer to Innes et al JDR 2016 caries terminology paper**

**R:** thanks to the reviewer for this suggestion. We considered the terminology referring to Innes et al (2016) Innes NP, Frencken JE, Schwendicke F. Don't Know, Can't Do, Won't Change: Barriers to Moving Knowledge to Action in Managing the Carious Lesion. J Dent Res. 2016 May;95(5):485-6. doi: 10.1177/0022034516638512. PMID: 27099269)

**Did you attempt to categorise the amount of crowding apart from 'low-middle'? Crowding features such as rotations and imbrications can have an effect on oral hygiene effectiveness**

**R:** thanks to the reviewer for this suggestion. Unfortunately, in this study, we included only patients with "low-middle" degree of crowding, in order to maintain the homogeneity between the two groups. Surely this inclusion criterion represents a limit for the present study. The reviewer with this observation gives us an indication to future studies that also include patients with “moderate/ severe” crowding. This was also already reported among the limits of the study at the end of the discussion section.

**Did you collect dietary data, as the type of appliance could influence dietary habits?**

**R:** unfortunately we have not investigated patients' eating habits. The reviewer with this observation gives us an indication to future studies.


**You do not discuss the limitations of the simplistic microbiological testing methodology you have used**

**R:** thanks to the reviewer for the suggestion. The following sentence was added in the Discussion: “*CRT® bacteria* was used to determine the *S. mutans* and *Lactobacilli* count in saliva by means of selective culture media. The preparation of samples and incubation were carried out according to the step-by-step procedure as it was described in its instruction brochure. This test only determines whether or not S.mutans are present in saliva in quantity that determines an increase of the caries risk.

The CRT package can be considered a comprehensive test, whose main benefits are to determine the caries risk status, to create the basis for target treatment and individualized check-up intervals for the long-term maintenance of oral health. This chair-side method is highly specific and sensitive for S. mutans and its only limitation is that at least 48 hours are required for detection of S. mutans.

Sánchez-García S, Gutiérrez-Venegas G, Juárez-Cedillo T, Reyes-Morales H, Solórzano-Santos F, García-Peña C. A simplified caries risk test in stimulated saliva from elderly patients. Gerodontology. 2008;25:26–33. doi:10.1111/j.1741-2358.2007.00184.x.

**Also, is salivary microbiome representative of the plaque microbiome? this should be discussed**

**R:** The literature is poor on this topic and an old study showed that there isn’t a direct correlation between the plaque amount and the saliva microbiome (Jalil RA. Correlating **Streptococcus mutans** counts in **saliva** with plaque amount, gingival inflammation and caries experience in school children. Singapore Dent J. 1995 Jul;20(1):16-20.).

Anyway both plaque and saliva microbiomes are associated with the development of dental caries. In the present study the evaluation of the PI, and the saliva microbioma, gives to the literature a new step in this field.

This point was added to the discussion following the suggestion of the reviewer.

**Are Lactobacilli involved in the early development of carious lesions?
R:** The evidence remains weak or absent about the association between initial dental caries and Lactobacilli. (Neves BG, Stipp RN, Bezerra DDS, Guedes SFF, Rodrigues LKA.Quantitative analysis of biofilm bacteria according to different stages of early childhood caries.Arch Oral Biol. 2018 Dec;96:155-161. doi: 10.1016/j.archoralbio.2018.09.007.

Periodontol 2000. 2016 Feb;70(1):128-41. doi: 10.1111/prd.12100. Salivary biomarkers for dental caries. Gao X, Jiang S, Koh D, Hsu CY)

On the other hand it seems that the early stage of caries is highly affected by mutans streptococci and visible dental plaque on maxillary incisors whereas cavities are strongly related to lactobacilli that are then involved in the lesions development (Parisotto TM, Steiner-Oliveira C, Duque C, Peres RC, Rodrigues LK, Nobre-dos-Santos M. Relationship among microbiological composition and presence of dental plaque, sugar exposure, social factors and different stages of early childhood caries.Arch Oral Biol. 2010 May;55(5):365-73.)

This was also added to the discussion in order to make more clear for the reader the involvement of each considered parameter.

**In the cut off values for bacterial CFUs, you cite 4 papers, none of which investigated bacterial counts with carious lesion development - do you think these are appropriate references?**

**R:** 3 out of 4 cited papers are previously published studies that uses the same microbiological technique and cutoff values for caries risk evaluation. Anyway, according to what suggested by the reviewer we removed these 3 citations and added another citation that observed the CFU cutoff value applied in the present study.

(Gábris K, Nagy G, Madléna M, Dénes Z, Márton S, Keszthelyi G, Bánóczy J.Associations between microbiological and salivary caries activity tests and caries experience in Hungarian adolescents. Caries Res. 1999 May-Jun;33(3):191-5.)

**Is there any evidence that salivary buffering capacity tested as you have done is correlated with caries risk?**

**R:** Salivary buffering capacity is correlated with caries risk as reported by different articles as Fernando S, Tadakamadla SK, Bakr M, Scuffham PA, Johnson NW. Indicators of Risk for Dental Caries in Children: A Holistic Approach. JDR Clin Trans Res. 2019 Oct;4(4):333-341. that used a similar caries risk analysis kit and was reported in the manuscript methods section.

The reference was also added to the manuscript.

**Did you examine the participants for carious lesions? if not - why not?
If you did, why isn't it reported?**

**R:** All subjects were examined for carious lesions and were free from caries before being included in the study and checked during the therapy.

We thank the reviewer for reminding us to include this as an inclusion criteria in the manuscript, it wasn’t previously reported because for every orthodontist this is a criteria for starting an orthodontic treatment and is considered a routine check.

Of the four references you use to justify the cut-off values for CFUs, only one correlated Lactobacilli counts to carious lesions, and the majority of children had cavitated lesions - which i assume is not the case in your study- these are not sound references to use with early carious lesions

**R:** As previosly stated, 3 out of 4 cited papers are previously published studies that uses the same microbiological technique and cutoff values for caries risk evaluation. Anyway, according to what suggested by the reviewer we removed these 3 citations and added another citation that observed the CFU cutoff value applied in the present study. (Gábris K, Nagy G, Madléna M, Dénes Z, Márton S, Keszthelyi G, Bánóczy J.Associations between microbiological and salivary caries activity tests and caries experience in Hungarian adolescents. Caries Res. 1999 May-Jun;33(3):191-5.)

Reviewer #2:

This manuscript described to investigate salivary levels of Streptococcus mutans (S.mutans) and Lactobacilli, and other salivary indices in patients wearing clear aligners (CA) versus multibrackets orthodontic appliances (MB).The authors determined Plaque index (PI), salivary flow, buffering power of saliva, and

salivary levels of S.Mutans and Lactobacilli were evaluated before the beginning of the orthodontic treatment (t0), and after 3 months (t1) and 6 months (t2) from the beginning. The authors recruited 80 subjects with equal numbers of Mb and CA treatments.

Overall this study is an interesting area of investigation; however, there were some limitations and questions that the author should clarify for the comprehensive understanding of the manuscript.

1. **Please provide the time of saliva collection since it affects the composition and flow of saliva.**

**R:** Saliva was collected during morning (9-12am). This data was added in the Material and Methods section.

**2. Please discuss the association between number of bacteria and incidence of decalcification in the discussion.**

**R:** Literature has not clarified yet, whether there is a direct proportion between the count of bacterial colonies in the saliva and the appearance/increase of decalcifications on the teeth during an orthodontic treatment. This correlation is actually derived from a clinical observation according to which decalcifications near to orthodontic brackets can increase during an orthodontic treatment (Morrier JJ. White spot lesions and orthodontic treatment. Prevention and treatment. Orthod Fr. 2014 Sep;85(3):235-44. doi: 10.1051/orthodfr/2014016. Epub 2014 Aug 28), and from the laboratory observation that the number of bacterial colonies correlates in the saliva is linked to a higher risk to develop enamel decalcification (Messer LB. Assessing caries risk in children. Aust Dent J. 2000;45:10–6.). While from one of the classical study in literature dated 1989, S.mutans has been detected in the dental plaque near an orthodontic band both in areas interested by enamel dissolution, than in other areas of the teeth not interested by enamel dissolution, without any relationship between the presence of S.mutans and enamel dissolution. (Boyar RM1, [Thylstrup A](https://www.ncbi.nlm.nih.gov/pubmed/?term=Thylstrup%20A%5BAuthor%5D&cauthor=true&cauthor_uid=2600252), [Holmen L](https://www.ncbi.nlm.nih.gov/pubmed/?term=Holmen%20L%5BAuthor%5D&cauthor=true&cauthor_uid=2600252), [Bowden GH](https://www.ncbi.nlm.nih.gov/pubmed/?term=Bowden%20GH%5BAuthor%5D&cauthor=true&cauthor_uid=2600252). The microflora associated with the development of initial enamel decalcification below orthodontic bands in vivo in children living in a fluoridated-water area.J Dent Res. 1989 Dec;68(12):1734-8.)

**3. Normally the orthodontic treatment will take about 12 months, would the author follow up after 6 months. If so, would the author observe any difference or trend of bacteria level?**

**R:** Patients were followed for orthodontic treatment beyond the period of this study. However, no salivary sample measurements were performed. Unfortunately, therefore, no data are available on the saliva microbiota beyond the period included in the follow-up of this study. Future research protocols could include this monitoring beyond the time of 6 months.

**4. Would the author explain the randomization process of subjects?**

**R:** Unfortunately no randomization was performed. The type of orthodontic treatment had been chosen by the clinician in agreement with the patient, but the subjects were included in the samples matching age and gender distributions as already reported in the materials and methods section.

Thank you for the opportunity to review this manuscript.
