# Supplementary material for: Salivary levels of Streptococcus mutans and Lactobacilli and other salivary indices in patients wearing clear aligners versus fixed orthodontic appliances: An observational study
Source: PLoS One. 2020 Apr 24;15(4):e0228798. doi: 10.1371/journal.pone.0228798 (PMC7182227; doi:10.1371/journal.pone.0228798)
Supplement: Attachment — Submitted filename: R2.docx [file pone.0228798.s004.docx]

Editor

Three main issues need to be addressed:

**1) Please consider more recent publications (Reviewer 2),**

We were happy to add the recent publication that was indicated by the reviewer 2 and was previously not included because it was published in November 2019 after the submission of this manuscript.

**2) Address concerns on study limitations and explanation on the limitations,**

Following the reviewers suggestions other study limitations principally involved with the absence of randomization and the use of the CRT system. Another limitation was the fact that only two bacterial species are mentioned

**3) Also since there are only 2 bacterial species mentioned, please explain how the results would be applicable to the overall microbiome (Reviewer 2).**

The conclusions were limited to the 2 considered bacterial species that are involved in caries development, while information about the overall microbiome are not obtainable from the results of the present study.

**4) The writing and organization of the manuscript need to be improved.**

**In the present new version of the manuscript, we tried to better organize the entire manuscript**

Thanks to the Editor for this suggestion. In this revised version of the manuscript we tried to improve the writing and the organization of the manuscript, mostly for the Discussion section, where the order of the paragraphs was changed. beginning the discussion with the data about the PI, and then about the other properties of saliva (the salivary flow and the buffering capacity) and finally discussing the data about the microbial colonization. Then limitations and conclusions were stated.

Reviewer #1: Thank you for your responses to our comments and questions. There are still outstanding issues with the paper

**The written English is still poor**

**R:** English was carefully revised.

**The examination for caries was raised - and you have included a statement that active caries was an exclusion criteria - how was this determined?**

**R:** “Regarding dental caries as an exclusion criteria, they were assessed by clinical examination with specillum and mirror before the beginning of the study, and during the study period at each appointment. In particular, the orthodontic treatment was not started (brackets or first aligner positionment) if caries were found at the first clinical examination, in that case, a conservative treatment of dental caries was performed before beginning the treatment. Consequently, no active caries were observed in the whole sample during the study period.”. This sentence regarding the caries management was added in the materials and methods section.

**You still have not included caries data in the paper - if you are relating bacterial counts to carious lesion development, you should present the caries data and correlate it to the bacterial counts**

**R:** As reported in the previous answer and now reported in the study, no caries were observed during the study period, for this reason no further caries data were reported. The relationship between carious lesion development and bacterial count come from the previous literature, in the present study, a 6 months follow-up in subjects with medium to very good oral hygiene (as related to exclusion criteria) did not show the development of carious lesions that anyway wasn’t one of the objectives of the study.

**Your comment regarding the validity of the CRT system may be challenged by microbiologists**

**R: Thanks to the reviewer for this comment.** The use of the CRT system was explicitely reported as a limitation of the study according to the suggestion of the reviewer. In particular, it was used because it is a method that every dentist can adopt in his/her daily clinical routine, to monitor his/her patients during orthodontic treatment in a very simple way, differently from microbiological tests that are more difficult to be used in the daily routine

**Regarding the Table s- i believe that several could be combined (such as Tables 6 and 7), or entered into the text (such as Table 4)**

**R:** Thanks to the reviewer for this suggestion. In the present version of the paper, table 4 was deleted, as its data were entered into the text, while tables 6 and 7 were combined

**Your conclusions state that the CA are at lower risk of caries development - you cannot conclude this as you have not provided caries data - all you can conclude is about what you have measured - two bacterial counts and saliva characteristics**

**R:** The conclusions were restructured deleting the sentence about dental caries and reporting only what observed about oral hygiene and bacterial count.

Reviewer #2:

**A recent article published in American Journal of Orthodontics and Dentofacial Orthopedics demonstrated the oral microbiome comparison between fixed, clear aligner and control groups and showed a different result from this current study. Please refer to the AJODO study and discuss about the different result so the manuscript will provide more information.**

**R:** We really thank the reviewer for showing us the publication of a very recent paper (November 2019) about oral microbiome in clear aligners treatment (Alterations of the Oral Microbiome in Patients Treated with the Invisalign System or with Fixed Appliances.” American journal of orthodontics and dentofacial orthopedics. 156.5 (2019): 633–640.). After a careful evaluation of the article that analyzed a much wider microbiome spectrum, they state that “changes in the microbiome associated with Invisalign treatment showed different effects on oral health, with some beneficial and some harmful” and in particular they observed some negative results about periodontal harmful bacteria. Regarding caries involved bacteria, perfectly in agreement with this study, they observed that “For the Invisalign group, the abundance of Firmicutes was less than the fixed appliance group (I vs F) with no difference than the control group (I vs C)” and Firmicutes represents the Phylum of both S. Mutans and Lactobacilli analyzed in this study, related with caries risk rather than periodontal aspects. Furthermore, the sample size of that study is small (7 subjects with aligners and 14 with fixed appliances) and it is probably underpowered, in fact, no sample power analysis was reported, thus, the trustworthiness of that study (especially when they state an absence of significant differences between the microbiota of the two groups) is limited, this could be the reason of its contrast with the most complete systematic review on this topic that showed a better periodontal health in subjects in orthodontic treatment with clear aligners.

For this reason, we discussed the results of that recent study regarding caries related bacteria (i.e. Firmicutes - S. Mutans and Lactobacilli) that are the outcome of our study in in the proper section.

**The selection bias could affect the result and conclusion. There was a reported relationship between oral hygiene status and socioeconomic status and attitude toward types of appliances. Please state the limitation of your subject recruitment in the discussion since there is no random sampling in the process.**

**R:** Thank you for the interesting observation, it was added to the limitations of the study.

**The conclusion should be rewritten to be specific to the species studied in the manuscript.**

**R:** The conclusions were rewritten in order to be specific to the outcomes of the present study.
